# Polyphenol Consumption and Its Association with Physical and Mental Health in Adults with Major Depressive Disorder

**DOI:** 10.3390/nu18010047

**Published:** 2025-12-22

**Authors:** Joanna Rog, Paulina Pawlikowska, Małgorzata Futyma-Jędrzejewska, Paulina Wróbel-Knybel, Ryszard Maciejewski, Kinga Kulczycka, Hanna Karakula-Juchnowicz

**Affiliations:** 1 Institute of Medical Science, John Paul II Catholic University of Lublin, Konstantynów 1 H Street, 20-708 Lublin, Poland; 2 Institute of Human Nutrition Sciences, Warsaw University of Life Sciences (SGGW-WULS), Nowoursynowska 159C Street, 02-776 Warsaw, Poland; pawlikowska09@wp.pl; 3 1st Clinical University Hospital in Lublin, Staszica 16 Street, 20-400 Lublin, Poland; malgorzatafutyma@interia.pl; 4 1st Department of Psychiatry, Psychotherapy and Early Intervention in Lublin, Medical University of Lublin, Głuska 1 Street, 20-059 Lublin, Poland; paulina.wrobel-knybel@umlub.edu.pl (P.W.-K.); hanna.karakula-juchnowicz@umlub.edu.pl (H.K.-J.); 5 Institute of Health Sciences, John Paul II Catholic University of Lublin, Konstantynów 1 H Street, 20-708 Lublin, Poland; ryszard.maciejewski@kul.pl (R.M.); kinga.kulczycka@kul.pl (K.K.)

**Keywords:** nutritional psychiatry, functional foods, phytonutrients, phytochemicals, lipids, metabolism, nutritional assessment, polyphenol

## Abstract

**Background/Objectives**: Research confirms that diet can influence the onset or course of depression. Polyphenols are bioactive plant compounds with proven beneficial effects on health. The aim of this study was to assess the relationship between polyphenol intake and the health status of individuals with major depressive disorder (MDD). **Methods**: The study included 44 participants. Health status was assessed using questionnaires adapted into Polish, body composition analysis, and laboratory blood tests. Polyphenol intake was estimated using the Phenol-Explorer program. **Results**: Among men, polyphenol intake was positively associated with glycated hemoglobin levels (R = 0.70; *p* = 0.038). Lower polyphenol intake in women was associated with poorer physical health (*p* = 0.014) and overall quality of life (*p* = 0.013). Polyphenol intake enhanced the effects of visceral fat content, muscle mass, severity of depressive symptoms (positive), and severity of stress symptoms (negative) on triglyceride levels. Polyphenol intake was positively associated with LDL cholesterol levels, and this relationship was attenuated by body water and fat content. Polyphenol intake weakened the relationship between fat content (negative) and quality of life (positive) with cortisol levels (R^2^ = 0.61; *p* < 0.001). **Conclusions**: Polyphenols act both directly and mediate the effects of other factors on the health status of individuals with MDD. Despite their proven beneficial effects, further research is needed to explore their potential impact and mechanisms of action in patients with MDD.

## 1. Introduction

Major depressive disorder (MDD) is an increasingly recognized mental disorder that poses both a health and economic challenge to the 21st century. According to World Health Organization (WHO), globally, an estimated 5% of adults suffer from depression [1]. MDD develops through a complex interaction of biological, psychological, and social factors, with pharmacotherapy and psychotherapy being the primary treatment strategies [1]. Approximately 30% of individuals with MDD do not achieve full remission after initiating pharmacotherapy with two or more first-line antidepressant treatments [2]. A growing body of scientific evidence indicates that nutrition may be an important factor determining the mental health. Nutrients can modulate the activity of many biological processes, including those found to be dysregulated in MDD, such as chronic low-grade inflammation, oxidative stress, gut microbiome changes, mitochondrial dysfunction, and hypothalamic–pituitary–adrenal (HPA) axis dysregulation [3]. An anti-inflammatory diet is a proposed strategy to improve health of people with MDD [4]. Foods with anti-inflammatory properties provide significant amounts of polyphenols. Polyphenols are divided into six representative groups: phenolic acids, flavonoids, stilbenes, tannins, coumarins, and lignans. The classification is based on both their function and chemical structure. Sources of polyphenols include fruits, vegetables, whole-grain cereals, wine, tea, coffee, nuts, and legumes [5]. The polyphenol content in some plant foods can reach up to 500 mg per 100 g of food. The total polyphenol intake in the general population has been estimated at approximately 0.9 g per day. The main sources of these compounds in the human diet are coffee, tea, wine, and fruits and vegetables (such as apples, oranges, and green beans), although this depends on the population and the geographical region examined [6,7].

Many studies emphasize that the amounts of polyphenols currently consumed in the diet are too low to exert beneficial effects on the human body [8]. Consequently, supplementation, the enrichment of foods with polyphenols, and the promotion of diets rich in their natural sources are gaining increasing importance [9]. Available data suggest that polyphenol supplementation may provide health benefits for individuals with MDD—both in terms of symptom severity and quality of life—and that higher polyphenol intake is inversely associated with depressive symptoms [10,11]. However, the current evidence base is still limited, and further studies in more homogeneous patient cohorts are needed, especially given the substantial heterogeneity of MDD.

Given the increasing interest in the role of polyphenols in the prevention and management of mental disorders, this study aimed to evaluate the dietary intake of polyphenols among individuals with MDD and to investigate their potential effects on health outcomes.

## 2. Materials and Methods

### 2.1. Participants

The study group consisted of 44 adult volunteers diagnosed with MDD according to the criteria of the Diagnostic and Statistical Manual of Mental Disorders, Fifth Edition (DSM-5) [12].

Inclusion criteria:

(1) Provision of written informed consent to participate in the study;
(2) Outpatients aged 18–60 years;
(3) Body mass index (BMI) between 18.5 and 35.0 kg/m^2^.

Exclusion criteria:

(1) Diagnosis of autoimmune, neurological, or immunodeficiency diseases; inflammatory bowel disease, irritable bowel syndrome, type 2 diabetes, cancer, and/or IgE-dependent allergies;
(2) Presence of co-occurring mental disorders (excluding personality disorders), including intellectual disability, organic brain injury, or addiction (excluding nicotine and caffeine);
(3) High suicide risk as assessed by the investigator;
(4) Infection within one month prior to the initial study visit;
(5) Adherence to a specific diet (e.g., elimination, plant-based, or weight-reducing diet);
(6) In the subgroup of women: pregnancy or lactation.

Prior to participation, all patients were informed about the objectives and procedure of the study, and were assured that they could withdraw from the study at any time without any consequences.

### 2.2. Data Sources and Outcome Measures

This study employed a desk research approach, which involves the analysis of previously collected and processed secondary data. The data were obtained from the SANGUT study (Study evaluating the effect of probiotic supplementation on mental status, inflammation, and intestinal barrier in major depressive disorder patients using a gluten-free or gluten-containing diet)—a prospective, randomized, placebo-controlled, double-blind, 12-week clinical trial [13]. The aim of the SANGUT study was to evaluate the effects of probiotic supplementation on mental health, inflammation, and intestinal barrier function in individuals with MDD following a gluten-free or a gluten-containing diet. The study was conducted in accordance with the Declaration of Helsinki and was approved by the Bioethics Committee of the Medical University of Lublin (KE-0254/104/2018) [14].

Based on the SANGUT study, the following data and research tools were used in this analysis:

(1) Socio-demographic and clinical data;
(2) Eating habits: Food Frequency Questionnaire (FFQ-6) and a three-day dietary record [15];
(3) Physical activity: International Physical Activity Questionnaire (IPAQ) [16];
(4) Anthropometric measurements: body weight, height, body mass index (BMI), and bioelectrical impedance analysis (BIA);
(5) Depressive symptom severity: Montgomery–Åsberg Depression Rating Scale (MADRS) and Beck Depression Inventory (BDI) [17,18];
(6) Quality of life: Short Form Health Survey (SF-36) [19];
(7) Perceived stress: Perceived Stress Scale (PSS-10) [20].

A detailed description of these instruments is provided in the protocol of the SANGUT.

Fasting venous blood samples were obtained from study participants between 8:00 and 10:00 a.m. The concentrations of the following biomarkers were determined:

(1) Stress marker: cortisol;
(2) Metabolic parameters: total cholesterol, low-density lipoprotein cholesterol (LDL-C), high-density lipoprotein cholesterol (HDL-C), triglycerides (TG), glucose, insulin, and glycated hemoglobin (HbA1c);
(3) Liver function indicators: alanine aminotransferase (ALT) and aspartate aminotransferase (AST).

Homeostasis model assessment of insulin resistance (HOMA-IR) was estimated based on fasting glucose and insulin concentrations.

#### Polyphenol Intake Assessment

The FFQ-6 questionnaire was used to assess long-term (12-month) polyphenols consumption [15]. The FFQ-6 evaluates the frequency of consumption of 62 food items divided into eight groups. Respondents were asked to indicate portion sizes.

Based on a 3-day dietary record (including two weekdays and one weekend day), short-term polyphenol intake was estimated. Data from the dietary questionnaires were entered into the Phenol-Explorer database—a comprehensive resource on the polyphenol content of foods [21,22,23]. The database includes information on approximately 500 different polyphenols derived from over 400 food sources [24]. Based on the estimated dietary polyphenol intake and a review of the available literature, participants were categorized into two groups: those with low (<2000 mg/day) and those with high (>2000 mg/day) polyphenol intake.

### 2.3. Statistical Analysis

Statistical analyses were performed using Statistica version 13.3 (StatSoft, TIBCO Software Inc., Palo Alto, CA, USA). The distribution of continuous variables was assessed using the Shapiro–Wilk test. Descriptive statistics were expressed as the mean ($\bar{x}$) and standard deviation (SD) for variables with an approximately normal (ex-Gaussian) distribution, and as the median (Me) and minimum and maximum values (Min–Max) for variables with a non-ex-Gaussian distribution.

For variables with an ex-Gaussian distribution, parametric tests were applied: Student’s *t*-test was used to assess differences between study groups (e.g., low vs. high polyphenol intake), and Pearson’s correlation matrix was used to examine relationships between continuous variables. For non-ex-Gaussian parameters, the Mann–Whitney U test and Spearman’s rank correlation coefficient (ρ).

Stepwise multiple regression analysis was conducted to determine the influence of several continuous predictors on the dependent variable. For all analyses, a *p*-value < 0.05 was considered statistically significant.

## 3. Results

Information about the study participants is presented in Table 1. The study included 44 individuals: 64% women (*n* = 28) and 36% men (*n* = 16), aged 18–59 years. The duration of illness was approximately three times longer in women than in men (*p* < 0.01), although no significant sex differences were observed in the number of psychiatric hospitalizations. Most participants (75%) were non-smokers. Women were also older than men (*p* < 0.01).

Polyphenol intake ranged from 1800 to 2400 mg/day. Fewer than half of the participants (*n* = 21) had a BMI within the reference range; 17 participants (39%) were overweight, and 6 (14%) were classified as having class I obesity. Half of the participants (*n* = 22) reported at least one comorbidity. The most frequently reported conditions included cardiovascular, respiratory, and gastrointestinal diseases.

Eighty-two percent of participants (*n* = 36) were taking medication—most commonly selective serotonin reuptake inhibitors (SSRIs; 41%), followed by mood stabilizers (23%) and serotonin–norepinephrine reuptake inhibitors (SNRIs; 20%). Ten percent of participants used tricyclic antidepressants. Information on medication use is presented in Figure S1, and on dietary supplements in Figure S2. Twenty-one participants (48%) reported taking dietary supplements, most commonly vitamin D (34%), magnesium (14%), and B vitamins (11%).

### 3.1. Polyphenol Intake

Long-term polyphenol intake data were obtained from 41 individuals, and short-term intake data from 32 individuals.

18 individuals (56%) consumed 1–2 g of polyphenols per day during the most recent period, 2 individuals (6%) consumed 0.5–1 g per day, and 12 individuals (38%) consumed 0–0.5 g per day.

Based on long-term intake data, 24 individuals (59%) consumed more than 2 g of polyphenols per day, 11 (27%) consumed 1–2 g per day, 5 (12%) consumed 0.5–1 g per day, and one individual (2%) consumed 0–0.5 g per day.

### 3.2. Polyphenol Intake and Health-Related Outcomes

Among the study participants, a significant relationship was observed between daily polyphenol intake (long-term) and certain blood biochemical parameters (*p* < 0.05). No association was found between polyphenols intake and blood biochemical parameters in women (*p* > 0.05). In the male group, a positive correlation was identified between overall (long-term) polyphenols intake and HbA1c levels (R = 0.695, *p* = 0.038).

No associations were observed between the amount of polyphenol in the diet and BMI or body composition parameters (*p* > 0.05). Furthermore, no associations were found between polyphenol intake and disease-related variables, including the presence or severity of disease symptoms (*p* > 0.05).

### 3.3. Polyphenol Intake and the Quality of Life

There was no association between polyphenol intake and the severity of stress symptoms (*p* > 0.05). Women with lower short-term polyphenol intake reported subjectively poorer physical health (based on SF-36 scale results) (*p* = 0.014) and overall quality of life (*p* = 0.013) compared with women with higher polyphenol intake. In men, no association was found between quality of life and dietary polyphenol intake (*p* > 0.05).

### 3.4. Predictive Models of Health Parameters Based on Polyphenol Intake

Stepwise regression analysis indicated that polyphenols (short-term intake in all cases) may be involved in modulating health-related physiological processes. The results of the proposed models are presented in Table 2, Table 3 and Table 4.

Polyphenol intake was found to modulate the effects of other examined parameters on TG concentrations. The proposed model explained 51.76% of the variance in TG concentrations among the study participants (R^2^ = 0.518, *p* < 0.001), with visceral adipose tissue, muscle mass, and the severity of depression and stress symptoms identified as significant predictors of TG levels.

Higher visceral adipose tissue, muscle mass, and severity of depression symptoms, as well as lower stress symptoms, were associated with increased TG concentrations. This effect may be further strengthened by higher polyphenol intake and attenuated by greater physical activity.

Short-term polyphenol intake was identified as a factor explained 28.02% of the variance in LDL cholesterol concentrations among the study participants (R^2^ = 0.280, *p* = 0.009), with body composition parameters modulating this relationship (Table 3). The number of polyphenols in the responders’ diet of was positively associated with LDL cholesterol concentration, and this effect may be attenuated by higher body water and fat content.

Quality of life (positive relationship) and body fat (negative relationship) were identified as significant predictors of cortisol levels, while short-term dietary polyphenol intake may modulate the strength of this relationship. The proposed model explained 61.35% of the variance in cortisol levels (R^2^ = 0.614, *p* < 0.001).

## 4. Discussion

The aim of this study was to examine associations between dietary polyphenol intake and health indicators in individuals with MDD. We found no significant associations with mental health measures (e.g., depressive symptoms, perceived stress), whereas physical health and health-related quality of life (SF-36) showed associations with polyphenol intake. In exploratory regression models, polyphenol intake was also associated with several biochemical markers (triglycerides, LDL cholesterol) and cortisol; these results should be interpreted as associations rather than causal effects, as no formal mediation or interaction analyses were performed.

MDD is a growing public health, socioeconomic, and personal burden, driven by a complex and not fully understood etiology [1]. To date, the causes have not been clearly identified, and the literature suggests a complex interplay between potential contributing factors [25]. Numerous studies have examined the relationship between nutrition and mood disorders. People with MDD tend to consume unhealthy, low-quality foods such as fast food and sweet or salty snacks, which consequently leads to lower consumption of fruits, vegetables, whole grains, and fish [26,27,28]. Recommended dietary changes for individuals with MDD include increasing the intake of foods rich in polyphenols, which are considered effective agents in the primary prevention and treatment of mental illnesses [29].

According to the results of our analysis of short-term polyphenol intake, the majority of participants (56%) consumed 1–2 g of polyphenols per day ($\bar{x}$ = 1910.2 ± 715.7 mg). Analysis of long-term intake showed that most respondents (59%) consumed more than 2 g per day ($\bar{x}$ = 2331.7 ± 1235.0 mg). Based on cohort study results, the highest polyphenol intake is recorded in Europe, ranging from 584 to 1786 mg/day [30]. The usual polyphenol intake among Polish adults is estimated at approximately 1 g/day, which is similar to that observed in other populations [31]. The high polyphenol intake in our study group may be the result of overestimation, which is common in long-term dietary assessments. Daily polyphenol intake varies depending on gender and age [8]. It has been reported that women tend to consume lower amounts of polyphenols compared with men [32]. The finding confirmed by our short-term intake results: women ($\bar{x}$ = 1884.4 ± 690.9 mg) versus men ($\bar{x}$ = 1966.9 ± 803.3 mg). Performed by us, the assessment of long-term polyphenol intake showed that women consumed a similar amount of polyphenols ($\bar{x}$ = 2406.7 ± 1131.2 mg) as men ($\bar{x}$ = 2214.4 ± 1412.8 mg, *p* > 0.05).

No association was found between polyphenol consumption and laboratory blood parameters, including insulin, glucose, HOMA-IR, ALT, AST, total cholesterol, and HDL cholesterol. A study conducted by Huang et al. (2017) reported that consumption of polyphenol-rich pomegranates did not lead to any significant changes in blood glucose levels, insulin concentrations, or HOMA-IR [33]. The lack of association between polyphenol intake and carbohydrate metabolism biomarkers was attributed to confounding factors in the study population, such as lifestyle and stimulant use [33]. In interventional studies assessing the effects of polyphenol consumption on liver function biomarkers, only some of the analyzed compounds (flavonoids and phenolic acids) demonstrated therapeutic effects. Future research should determine not only the optimal dose but also the specific polyphenol group and route of administration, as these factors significantly affect absorption and bioavailability [34].

Dietary polyphenols regulate lipid metabolism by stimulating lipolysis and inhibiting lipogenesis. They also suppress the expression of adipogenic genes, enhance fatty acid β-oxidation, and modulate the SIRT1/AMPK pathway, collectively improving lipid homeostasis and reducing fat accumulation. Despite these mechanisms, no significant association was found between polyphenol consumption and blood lipid profiles, as confirmed by the meta-analysis conducted by Wang et al. [34]. Polyphenols from black tea (one of the main sources of polyphenols in the Polish diet) did not lead to changes in total or LDL cholesterol concentrations, suggesting that the relationship between flavanols and lipid parameters is generally weak [7]. In contrast, Vitale et al. (2017) reported that individuals in the highest tertile of polyphenol intake exhibited more favorable lipid profiles, characterized by higher HDL cholesterol and lower LDL cholesterol and TG levels [35]. Interestingly, another study reported that individuals with higher total cholesterol and TG levels tended to consume greater amounts of polyphenols. Among those with dyslipidemia, phenolic acids and lignans were the main subclasses of polyphenols consumed. These conflicting findings may reflect differences in study populations, dietary sources of polyphenols, the relative proportions of specific polyphenol classes, and the presence of other bioactive compounds in the diet. Moreover, excessive intake of polyphenol-rich fruits (particularly those high in fructose) may contribute to hypertriglyceridemia in susceptible individuals [36].

No statistically significant association was observed between dietary polyphenol intake and BMI or body composition parameters, including fat mass, muscle mass, body water content, and visceral adipose tissue. To date, evidence regarding the effects of polyphenols on body composition has been inconsistent. An interventional clinical trial investigating polyphenol-enriched foods reported changes in body composition, specifically a reduction in body fat, but no significant effect on BMI [37].

In our study population of individuals with MDD, no association was found between polyphenol consumption and clinical course of MDD (duration of illness and number of psychiatric hospitalizations). Godos et al. (2018) [11] reported similar findings in their observational study, showing no correlation between the consumption of specific polyphenol-rich food groups (fruits and vegetables) and either the diagnosis or severity of depressive symptoms. However, the intake of specific subclasses (phenolic acids, flavanones, and anthocyanins) was inversely associated with depressive symptoms [11]. Therefore, analyzing the types of polyphenols present in the diet may be necessary to assess these relationships more precisely. In an intervention study involving young mothers, the impact of polyphenol supplementation (through the addition of at least one flavonoid-rich food to the participants’ habitual diet) on mood, anxiety, and quality of life was assessed. No differences were observed between the flavonoid-supplemented group and the control group after two weeks of intervention [38]. A short period of increased flavonoid consumption seems unlikely to exert measurable effects on mental health.

In MDD, abnormal HbA1c values relative to reference ranges have been reported, appearing to be associated with a more severe course of disease. Our study demonstrated a positive association between HbA1c concentration and polyphenol consumption (R = 0.6954; *p* = 0.038). Numerous studies have examined the effect of polyphenols on metabolic control, particularly HbA1c [39,40]. Most interventional studies indicate that polyphenols may have a preventive effect on the development of diabetes. The hypoglycemic effect of polyphenols may result from reduced intestinal carbohydrate absorption, inhibition of hepatic glucose release, and enhanced peripheral glucose uptake. In our study, the association between polyphenol intake and HbA1c was observed only in men. This could reflect reverse causality, a phenomenon in cross-sectional studies where observed relationships may be opposite to expected [41]. Overweight adults with metabolic risk, for example, are more likely to follow healthier diets [30,42]. Meta-analysis by Palma-Duran et al. (2017) [39] reported that polyphenol supplementation reduced HbA1c only in individuals with diabetes, with no effect in those without diabetes or with prediabetes, while Kosmalski et al. (2022) [43] found that polyphenols (except stilbenes) did not significantly alter HbA1c. In our study, diabetes was an exclusion criterion, and participants’ HbA1c values remained within the reference range [39,43].

A sex-specific relationship was observed between polyphenol intake and quality of life. Women who consumed lower amounts of polyphenols reported poorer physical health (*p* = 0.014) and lower overall quality of life (*p* = 0.013) compared with those consuming higher amounts. An 8-week intervention aimed at increasing dietary polyphenol intake significantly improved quality of life, regardless of gender. Barfoot et al. (2021) reported similar findings in their study on the mental health of young mothers [38]. They observed a positive effect of increased polyphenol consumption on the physical health domain of quality of life, but no significant association with its mental health dimension. Conversely, a study in individuals with hypertension did not find significant changes in anxiety, stress, or self-esteem among participants with higher polyphenol intake [44]. Although our study was observational and could not establish causality, these results—together with evidence from interventional studies—suggest that polyphenol consumption may influence the health and quality of life of individuals with MDD. One potential mechanism to explain the observed results in the aforementioned studies is the attenuating effect of polyphenols on neuroinflammation. Modulation of neuroinflammatory pathways may hypothetically represent one of the key mechanisms underlying these associations. Neuroinflammation in the central nervous system may differ from peripheral inflammatory processes, and future studies should aim to assess the direct effects of polyphenols on neuroinflammatory pathways within the brain, rather than relying solely on peripheral markers [45]. Given that the diet and inflammation are tightly linked in MDD, integrating biomarkers of immune activation in this study could have added depth to the interpretations [46].

Our results suggest that polyphenols may modulate—either enhancing or attenuating—the relationships between various health factors in individuals with MDD. Higher TG concentrations were associated with greater visceral fat and muscle mass, and higher polyphenol consumption appeared to potentiate this relationship. Excessive visceral fat accumulation is a well-known factor contributing to elevated TG concentrations [47]. These findings may seem unexpected, given the well-documented beneficial effects of polyphenols on lipid metabolism reported in the literature. Some anthocyanins are more readily absorbed from fruits with higher amount of sugar, whereas excessive dietary fructose promotes fat accumulation and disrupts lipid metabolism [48,49]. The subclasses of polyphenols consumed by participants were not assessed in this study; therefore, this relationship requires further investigation.

In our investigation, polyphenol intake was positively associated with LDL cholesterol levels. The relationship between dietary polyphenol intake and LDL cholesterol concentrations was attenuated by higher body fat and water content. This association within the study group may be attributable to the primary dietary source of polyphenols. Coffee contains high amounts of polyphenols, and as demonstrated by Surma et al. (2023), the type and preparation method of coffee can significantly affect the lipid profile [50]. A meta-analysis of clinical trials conducted by Schoeneck and Iggman (2021) compared the effects of filtered and unfiltered coffee on blood lipid parameters [51]. Consumption of unfiltered coffee was found to increase serum LDL cholesterol concentrations compared with filtered coffee. Caffeine in coffee may enhance lipolysis and increase LDL cholesterol levels, and this effect was not observed with decaffeinated coffee consumption [52]. The diuretic effect of high-caffeine coffee has been demonstrated, although some studies suggest that this effect is modest. Caffeine-induced diuresis was found to be six times greater in women. This sex-related variability may result from factors influencing caffeine metabolism (body weight and nutritional status) as well as differences in hormonal profiles [52,53]. Men generally have greater body mass and a higher proportion of body fat, which may mitigate dehydration associated with coffee consumption.

Our study demonstrated that cortisol levels in patients with MDD are influenced by body fat content (inverse relationship) and quality of life (positive relationship), and that this effect may be attenuated by higher dietary polyphenol intake, as well as higher subjective stress and depression severity. Similar findings were reported by Schorr et al. (2015), who observed that cortisol levels were lowest in women with overweight and class I obesity, and increased with extreme obesity or underweight [54]. Low body fat (underweight) is believed to activate the HPA axis, which may consequently contribute to muscle atrophy [54]. It is worth noting that individuals with extreme BMI values (≤18.5 kg/m^2^ or ≥35.0 kg/m^2^) were excluded from our study.

Several limitations of our study should be mentioned. The study population was relatively small, and including a larger number of participants could potentially reveal different results and associations. The strict inclusion and exclusion criteria were established to maintain group homogeneity and minimize the potential influence of confounding factors (such as concomitant diseases or BMI) on the results. Another limitation is the cross-sectional design of the study. Although polyphenol intake was assessed twice (recent consumption and habitual intake over the previous year), other variables were measured only once. Moreover, subclasses of polyphenols were not analyzed. The polyphenol composition of foods is highly variable and depends on the plant species, degree of ripeness, as well as processing and storage methods. Nutritional studies face numerous challenges; for example, collinearity between polyphenols and other dietary components may complicate data interpretation. No control group was included in this study. Examining only individuals with MDD limits the contextualization of our findings, making it difficult to determine whether the observed associations are specific to this population or reflect more general patterns. An exploratory descriptive research design was implemented to characterize the physiological and psychological factors among individuals with MDD. It should be emphasized that this study was observational in nature and represents a secondary analysis; the polyphenol analysis was never pre-specified, and the dataset was not originally designed for this purpose. Nevertheless, dietary intake data were collected individually and meticulously by an experienced dietitian, which is uncommon in large epidemiological studies and helps minimize recall bias. Therefore, the aim was not to establish causal relationships between the examined variables. Another important limitation is the restricted scope of data analysis due to the small sample size, which may lead to unstable or overfitted estimates and increase the risk of false-positive associations. Therefore, all findings should be considered preliminary and exploratory rather than indicative of definitive biological effects. Although standardized and meticulous data collection procedures enhance the reliability of the results and help mitigate recall bias, the limited sample size remains a constraint. Factor-based analyses can nevertheless provide meaningful insights in smaller samples, particularly when variables are biologically and behaviorally related and the analyses are used for data reduction and pattern exploration rather than for definitive inference. While small sample sizes and stepwise regression approaches may offer certain advantages in exploratory research, they are also associated with important limitations that may affect the robustness and generalizability of the results. Accordingly, the findings should be viewed as hypothesis-generating rather than confirmatory.

Despite limitations, the research provided a comprehensive examination of numerous physiological and psychological parameters, enabling a more precise and in-depth characterization of the potential associations between dietary polyphenol intake and clinical manifestations of MDD. Although the study does not fully address the multifaceted nature of the disorder, given its complex and not yet fully elucidated etiology, the findings represent an important contribution to the growing body of evidence on the role of polyphenols within the field of nutritional psychiatry.

## 5. Conclusions

(1) A positive association was observed between dietary polyphenols and HbA1c in men. This result may potentially reflect gender differences in the metabolism of polyphenolic compounds or their influence on carbohydrate metabolism. The effect could also be modulated by other dietary or metabolic factors not considered in this study.
(2) A positive association was found between the number of polyphenols consumed and LDL cholesterol concentration. This may suggest a complex and context-dependent effect of polyphenols on lipid profiles, potentially influenced by the type and source of phenolic compounds, their bioavailability, and concomitant dietary and environmental factors.
(3) Polyphenols may play a modulating role in certain aspects of health. Higher polyphenol consumption appeared to strengthen the relationship between selected factors and TG concentrations, while potentially weakening relationships between specific variables and cortisol levels. These findings could indicate a modulatory effect of polyphenols on mechanisms regulating lipid metabolism and the body’s stress response, but they should be interpreted with caution.
(4) No significant relationship was observed between polyphenol consumption and clinical outcomes in the study population. This may indicate that their impact on disease progression is limited or dependent on other biological and environmental factors.
(5) In the group of women, a significant association was demonstrated between the amount of polyphenol consumption and subjectively assessed quality of life. Lower polyphenol consumption was associated with poorer physical health and lower overall quality of life.
(6) Further research is needed to clarify which types of polyphenols may exert the most beneficial effects on specific health parameters and how these compounds interact with other modifiable lifestyle and environmental factors.

## Figures and Tables

**Table 1:** Characteristics of the examined population.

Factor	Sex	$\bar{x}$ /Me	SD/Min.–Max.	*p*-Value
Age [years]	K	43.9	9.2	<0.010 *^a^
	M	30.8	10.3	
	Total sample	39.2	11.7	
BMI [kg/m^2^]	K	25.8	3.9	0.793 ^a^
	M	25.5	3.3	
	Total sample	25.7	3.7	
Duration of illness [months]	F	120.0	2.0–492.0	<0.010 *^b^
	M	24.0	3.0–216.0	
	Total sample	60	2.0–492.0	
Number of psychiatric hospitalizations	F	0	0–6.0	0.440 ^b^
	M	0	0–2.0	
	Total sample	0	0–6.0	
Polyphenol intake [short-term, mg/day]	F	1884.4	690.9	0.632 ^a^
	M	1966.9	803.3	
	Total sample	1910.2	715.7	
Polyphenol intake [long-term, mg/day]	F	2406.7	1131.2	0.767 ^a^
	M	2214.4	1412.8	
	Total sample	2331.7	1235.0	

$\bar{x}$—mean; Me—median; SD—standard deviation; Min.—minimum; Max.—maximum; *—statistical significance; ^a^—Student’s *t*-test (for independent groups); ^b^—Mann–Whitney U test; F—females; M—males. Bold indicates statistical significance (*p* < 0.05).

**Table 2:** Regression analysis with triglycerides (TG) as the dependent variable.

Independent Variables	β	*p*-Value
Visceral fat tissue [%]	0.546	<0.001 *
Muscle mass [%]	0.420	0.004 *
Physical activity [IPAQ]	−0.181	0.196
Depressive symptoms [BDI]	0.715	0.006 *
Stress severity [PSS-10]	−0.635	0.013 *
Polyphenol intake [short-term, mg/day]	0.191	0.188

β—standardized regression coefficient; *—statistical significance; IPAQ—International Physical Activity Questionnaire; BDI—Beck Depression Inventory; PSS-10—Perceived Stress Scale. Bold indicates statistical significance (*p* < 0.05).

**Table 3:** Regression analysis with LDL cholesterol as the dependent variable.

Independent Variables	β	*p*-Value
Total body water [%]	−1.668	0.145
Polyphenol intake [short-term, mg/day]	0.414	0.015 *
Fat tissue [%]	−1.216	0.283

β—standardized regression coefficient; *—statistical significance. Bold indicates statistical significance (*p* < 0.05).

**Table 4:** Regression analysis cortisol as the dependent variable.

Independent Variables	β	*p*-Value
Fat tissue [%]	−0.693	<0.001 *
Stress severity [PSS-10]	−0.352	0.193
Quality of life [SF-36]	0.458	0.040 *
Polyphenol intake [short-term, mg/day]	−0.231	0.089
Depressive symptoms [BDI]	−0.411	0.110

β—standardized regression coefficient; *—statistical significance; PSS-10—Perceived Stress Scale; SF-36—36-Item Short Form Health Survey; BDI—Beck Depression Inventory. Bold indicates statistical significance (*p* < 0.05).

## Data Availability

The original contributions presented in this study are included in the article/Supplementary Materials. Further inquiries can be directed to the corresponding author.

## Supplementary Materials

The following supporting information can be downloaded at: https://www.mdpi.com/article/10.3390/nu18010047/s1, Figure S1: Overview of medication use among study participants. Figure S2: Overview of dietary supplement use among study participants.

## Abbreviations

The following abbreviations are used in this manuscript:

MDD	Major Depressive Disorder
WHO	World Health Organization
DSM-5	Diagnostic and Statistical Manual of Mental Disorders, Fifth Edition
BMI	Body Mass Index
IgE	Immunoglobulin E
HPA	Hypothalamic–Pituitary–Adrenal axis
FFQ-6	Food Frequency Questionnaire (version 6)
IPAQ	International Physical Activity Questionnaire
BIA	Bioelectrical Impedance Analysis
MADRS	Montgomery–Åsberg Depression Rating Scale
BDI	Beck Depression Inventory
SF-36	36-Item Short Form Health Survey
PSS-10	Perceived Stress Scale (10-item version)
SANGUT	Study evaluating the effect of probiotic supplementation on mental status, inflammation, and intestinal barrier in MDD patients using a gluten-free or gluten-containing diet
ALT	Alanine Aminotransferase
AST	Aspartate Aminotransferase
LDL-C	Low-Density Lipoprotein Cholesterol
HDL-C	High-Density Lipoprotein Cholesterol
TG	Triglycerides
HbA1c	Hemoglobin A1c (Glycated Hemoglobin)
HOMA-IR	Homeostasis Model Assessment of Insulin Resistance
SSRIs	Selective Serotonin Reuptake Inhibitors
SNRIs	Serotonin–Norepinephrine Reuptake Inhibitors
TCAs	Tricyclic Antidepressants
$\bar{x}$	Mean
Me	Median
SD	Standard Deviation
Min	Minimum
Max	Maximum
