# Supplementary material for: Polyphenol Consumption and Its Association with Physical and Mental Health in Adults with Major Depressive Disorder
Source: Nutrients. 2025 Dec 22;18(1):47. doi: 10.3390/nu18010047 (PMC12788040; doi:10.3390/nu18010047)
Supplement: Supplementary file 1 [file nutrients-18-00047-s001.zip › nutrients-4006329-supplementary.pdf]

Supplementary Data

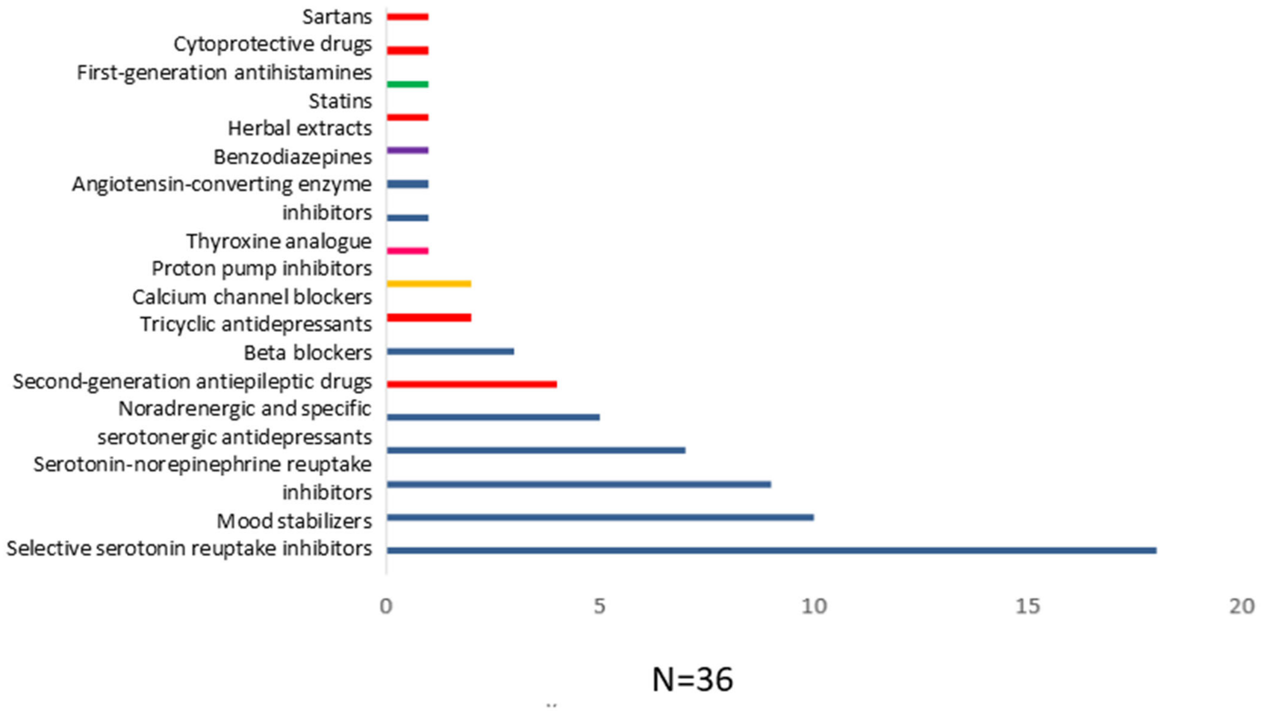

Figure S1. Overview of medication use among study participants.

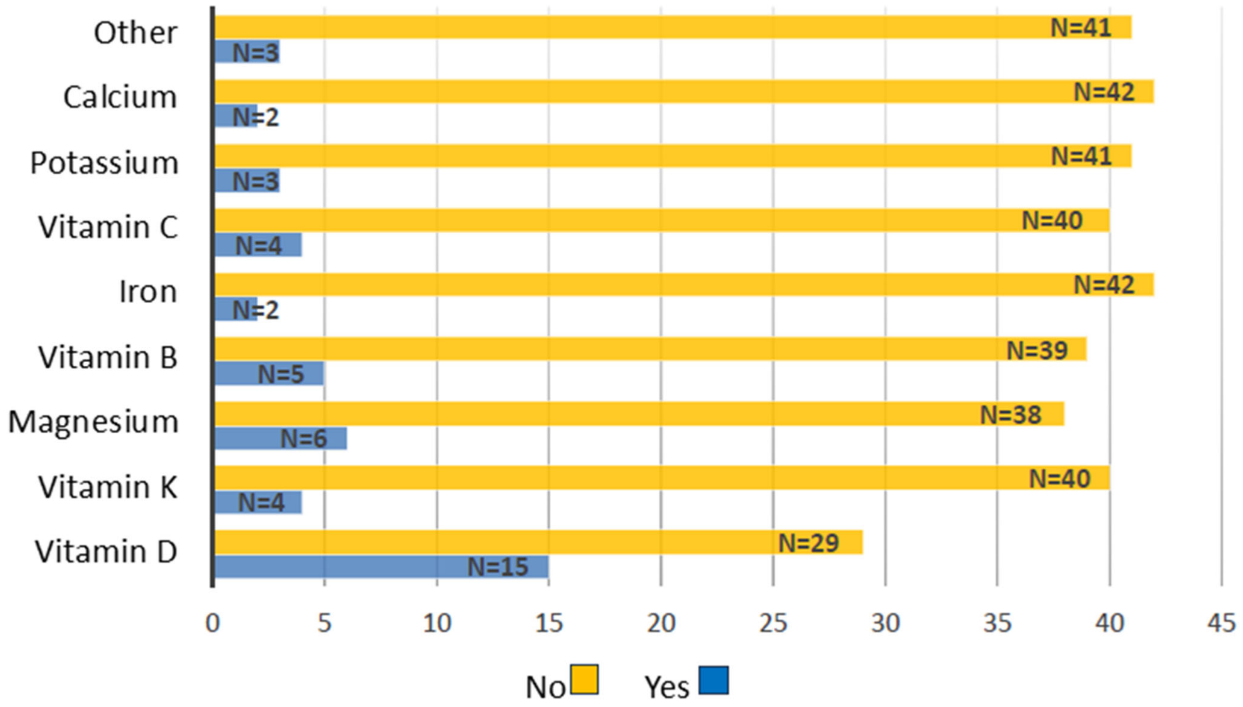

Figure S2. Overview of dietary supplement use among study participants.
